# The spatial variation and driving factors of soil total carbon and nitrogen in the Heihe River source region

**DOI:** 10.1007/s10661-023-11251-4

**Published:** 2023-05-25

**Authors:** Shan Tong, Guangchao Cao, Zhuo Zhang, Jinhu Zhang

**Affiliations:** 1 School of Geographical Sciences, Qinghai Normal University, Xining, 810008 Qinghai China; 2 Qinghai Provincial Key Laboratory of Physical Geography and Environmental Processes, Qinghai Normal University, Xining, 810008 Qinghai China; 3 Ministry of Education Key Laboratory of Qinghai-Tibet Plateau Surface Processes and Ecological Conservation, Xining, 810008 Qinghai China; 4 Xinjiang Kezhou Environmental Monitoring Station, Kezhou, 845350 Xinjiang China; 5 Xi’an, Shaanxi Province China; 6 Cangshan, Shandong Province China

**Keywords:** RDA analysis, Horizontal distribution, Vertical distribution, Different land use types, Heihe River source region

## Abstract

Soil carbon and nitrogen levels are key indicators of soil fertility and are used to assess ecological value and safeguard the environment. Previous studies have focused on the contributions of vegetation, topography, physical and chemical qualities, and meteorology to soil carbon and nitrogen change, but there has been little consideration of landscape and ecological environment types as potential driving forces. The study investigated the horizontal and vertical distribution and influencing factors of total carbon and total nitrogen in soil at 0–20 and 20–50 cm depths in the source region of the Heihe River. A total of 16 influencing factors related to soil, vegetation, landscape, and ecological environment were selected, and their individual and synergistic effects on the distributions of total carbon and total nitrogen in soil were assessed. The results show gradually decreasing average values of soil total carbon and total nitrogen from the surface layer to the bottom layer, with larger values in the southeast part of the sampling region and smaller values in the northwest. Larger values of soil total carbon and total nitrogen at sampling points are distributed in areas with higher clay and silt and lower soil bulk density, pH, and sand. For environmental factors, larger values of soil total carbon and total nitrogen are distributed in areas with higher annual rainfall, net primary productivity, vegetation index, and urban building index, and lower surface moisture, maximum patch index, boundary density, and bare soil index. Among soil factors, soil bulk density and silt are most closely associated with soil total carbon and total nitrogen. Among surface factors, vegetation index, soil erosion, and urban building index have the greatest influence on vertical distribution, and maximum patch index, surface moisture, and net primary productivity have the greatest influence on horizontal distribution. In conclusion, vegetation, landscape, and soil physical properties all have a significant impact on the distribution of soil carbon and nitrogen, suggesting better strategies to improve soil fertility.

## Introduction

Soil carbon and nitrogen are key parameters involving soil quality, nutrient supply, biogeochemical cycles, and global climate change (Bai et al., 2017; Galloway et al., 2008). Nitrogen content in soil is a significant factor affecting vegetation growth. Plants primarily absorb nitrogen from the soil in the form of nitrate nitrogen to synthesize essential substances (Geng et al., 2011). The soil carbon pool plays a vital role in the research on carbon cycling and carbon balance in terrestrial ecosystems (Yu et al., 2005). The soil carbon pool reflects the trend of the global carbon cycle, including carbon storage and greenhouse gas emissions (Tong, 2020). Given growing concerns about global climate change, there is significant interest in understanding the variation characteristics of soil carbon content and its influencing factors (Huang et al., 2020).

The source region of the Heihe River is in a frigid zone with an extremely fragile ecological environment and a low natural recovery ability (Diao, 2019). Most (87.5%) of the source region of the Heihe River is in Qilian County (Tian et al., 2014). This vital water supply area is part of the Qilian Mountains National Park, and changes in this region can affect the environmental quality of the Heihe River. Soil is a multi-phase complex system, and changes in soil carbon and nitrogen involve many interrelated biochemical processes, such as vegetation type, climate change, soil physical and chemical properties, and natural disasters, such as floods and soil erosion (Andriamananjara et al., 2016; Feng et al., 2013; Wang et al., 2002; Zhang et al., 2009a, b). There have been a series of ecological construction and protection projects in this region, including the natural forest protection project, the control of waters, forests, farmland, lakes, and grassland project, and the grain for green project. Previous studies on the influencing factors of soil total carbon and nitrogen mainly focused on vegetation (Yu, 2020; Tong et al., 2022), topography (Hu et al., 2006; Wu et al., 2022; Zhai et al., 2019), physical and chemical properties (Qu, 2019; Huang et al., 2020; Diao, 2019; Bian et al., 2018), and meteorology (Han et al., 2013; Yuan et al., 2018; Zhang et al., 2009a, b), but the potential contributions of the landscape and ecological environment indicators have not been explored. Additionally, few comprehensive studies have been conducted, so the main influencing factors of soil quality in this important region have not been determined.

In this study, field sampling, indoor experiments, and remote sensing data analyses are conducted to assess the total carbon and total nitrogen content in soil and physicochemical property indicators at different soil depths. The horizontal and vertical distributions of TC and TN in soil were determined at 0–20 and 20–50 cm depths. Finally, the individual and synergistic effects of multiple influencing factors on the spatial pattern of soil TC and TN for a comprehensive study of this critical region.

## Overview of research area

**Fig. 1:**
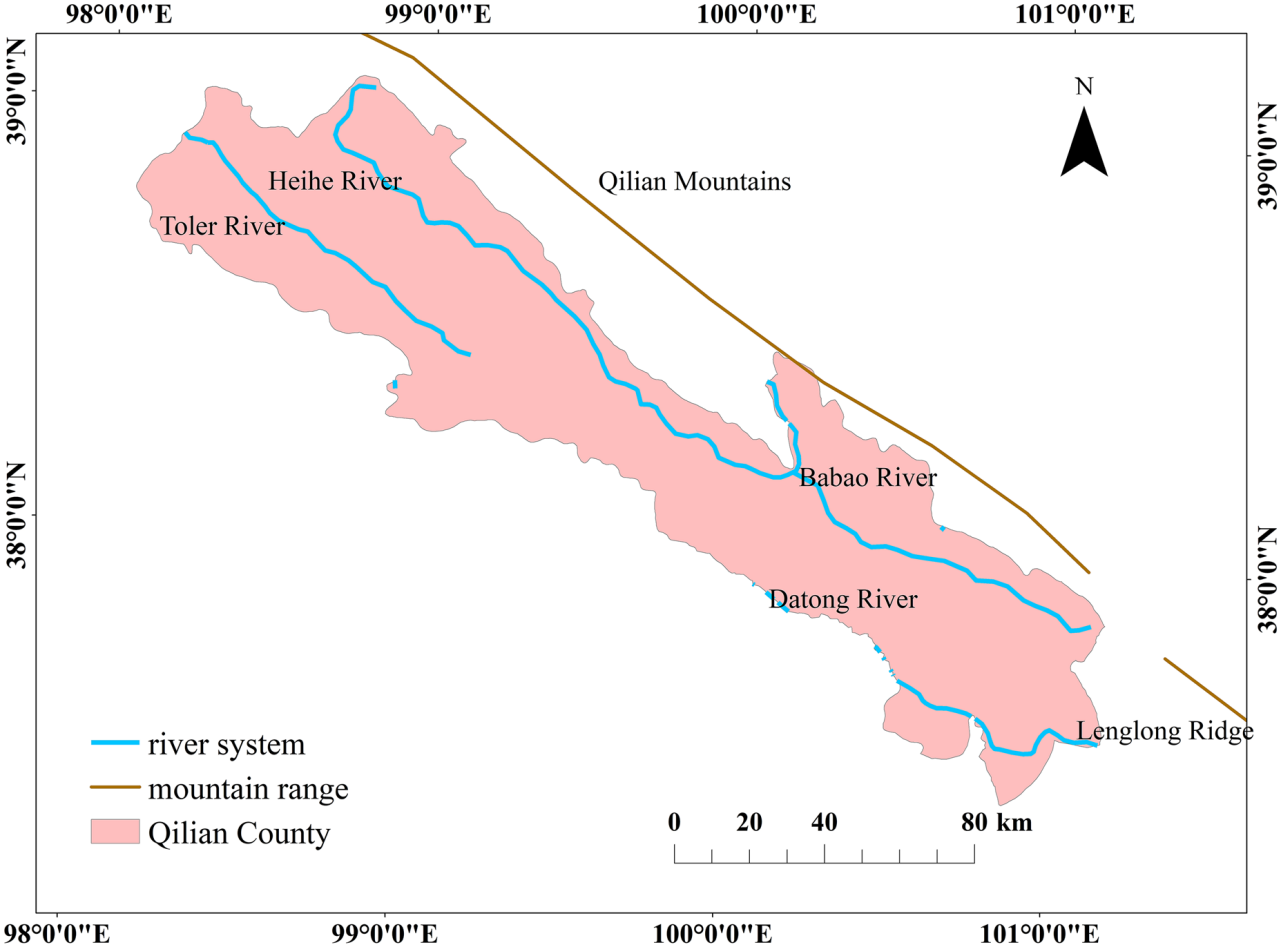
Geographical map of the study area

Qilian County is in northeastern Qinghai Province and northwest Haibei Tibetan Autonomous Prefecture, in the middle of the Qilian Mountains (Fig. 1). The county includes 14,000 km^2^ and ranges in altitude from 2646 to 5264 m, with an average of 3500 m (Tian et al., 2014). Qilian is a key ecological security barrier in China, connected to the Hexi Corridor in the north and the Qinghai Lake’s transit channel in the south. Qilian is in the “north line” of the province’s “one circle and three lines” tourism strategy layout and serves as a transportation hub and gateway to the outside world (Zhang et al., 2021). The region is considered an important “gene bank” for cold-zone species (Ma et al., 2021). The plateau continental climate region has an annual average temperature of 1 °C and annual precipitation of 420 mm (Ma et al., 2021). There are several rivers in Qilian County: the Heihe River, China’s second largest inland river; the Datong River, a Yellow River tributary; and the Tuole River, Jiayuguan, Gansu Province’s mother river. These many rivers make this region an important water source conservation area and a valuable ecological barrier in western Gansu, with irreplaceable ecological functions (Yuan, 2019).

## Data sources and methods

### Data sources

#### Soil data

**Fig. 2:**
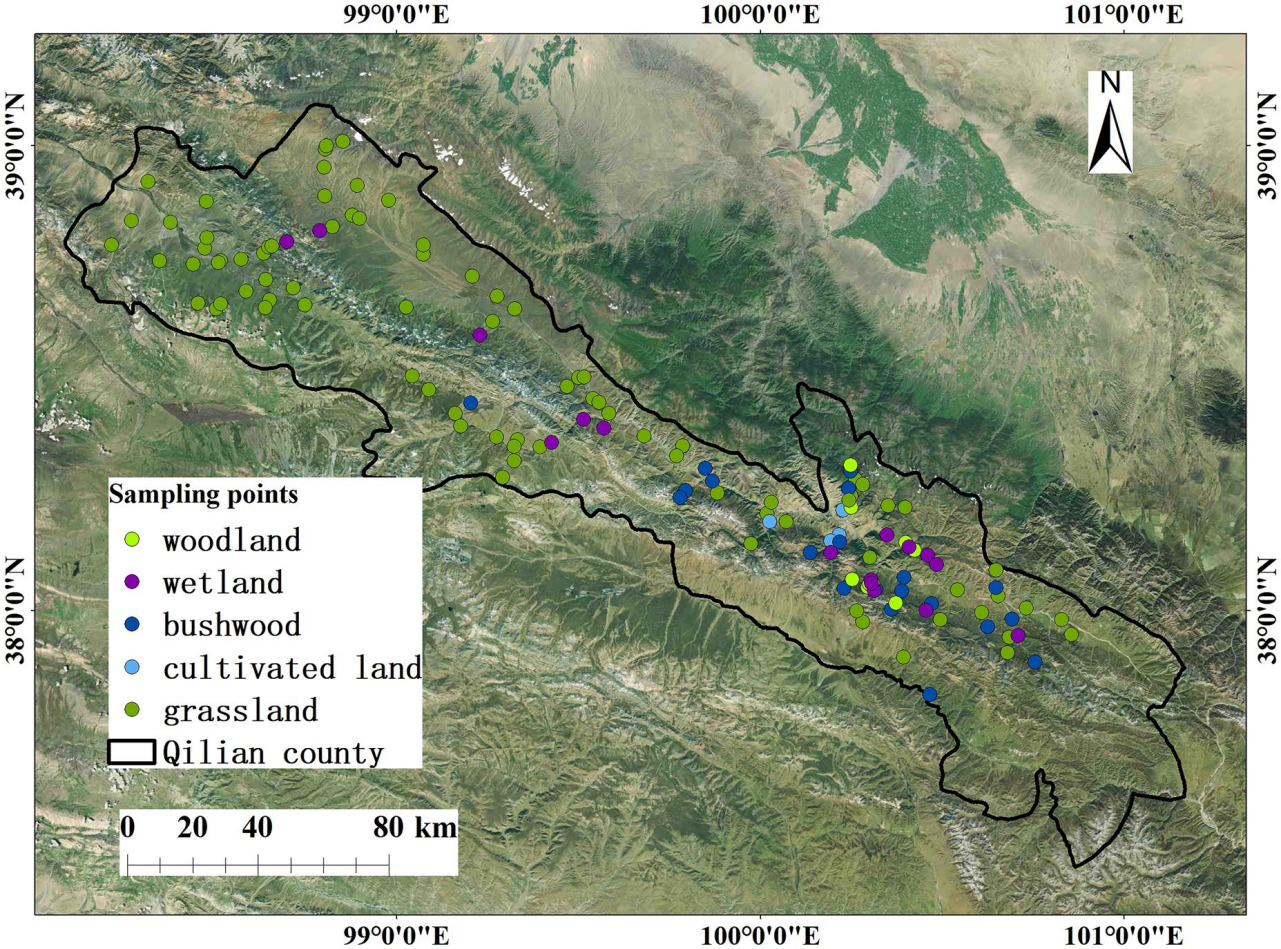
Distribution of sampling points in different ecosystems

The root systems of plants in the frigid grassland in the Qilian Mountains are primarily concentrated in the surface layer. Some shrub root systems start to grow vertically downward at the surface layer but switch to grow horizontally after a certain depth (Yang et al., 2007). Therefore, the nutrient pool of surface soil is the main supply of plant mineral nutrients (Yang et al., 2008). According to the stratification by physical properties of soil in the Qilian Mountains and the Three-River-Source region, Liu et al. (2021), He et al. (2006), and Yang et al. (2008) classified the soil layer at 0–20 cm as the surface soil and the soil layer at 20–50 cm as the deep soil. The woodlands on the southern slope of the Qilian Mountains are dominated by spruce and juniper; the shrubs are dominated by Potentilla fruticosa (Shrubby Cinquefoil), Dasiphora davurica, and thorns; and the grasslands are dominated by Achnatherum splendens. In the study area, grassland accounts for 54.57% of the total area, bushwood accounts for 12.07%, forest accounts for 3.31%, and wetland accounts for 9.31%. Sampling was carried out based on traffic accessibility and the need to sample different altitudes and ecosystems in the study area. For each type of plot, 20 m × 20 m quadrats were set up. In each plot, three quadrats sized 1 × 1 m were randomly arranged, and soil samples at 0–50 cm were drilled with a soil drill with a diameter of 5 cm. We carried out the sample according to the national standards (technical specification for soil environmental monitoring, HJ/T 166–2004; guidelines for the design of soil sampling procedures for soil quality, GB/T 36199–2018). The sampling time, location, vegetation coverage, and orientation were recorded. In each sample plot, holes with a diameter of 5 cm were drilled, and soil samples from 0–10, 10–20, 20–30, 30–40, and 40–50 cm soil layers were collected. After the removal of gravel and grassroots, samples were mixed and air-dried before nutrient testing. The latitude and longitude, vegetation coverage, altitude, and ecosystem type of each sampling point were recorded. A total of 705 samples from 141 sampling points were collected in August 2018, including 92 grassland samples, six woodland samples, five cultivated land samples, 21 bushwood samples, and 17 wetland samples (Fig. 2).

#### Image data

Landsat8 data, with a spatial resolution of 30 × 30 m, were obtained from http://www.gscloud.cn/. For the images from August 2018, Fragstats v4.2.1 was used to calculate landscape indicators. For NDVI data, vegetation remote sensing data MOD13Q1, MOD09A1, and MOD17A3HGF were selected from the NASA website (https://ladsweb.modaps.eosdis.nasa.gov/). The spatial resolution was 250 × 250 m or 500 × 500 m, and the temporal resolution was 16 or 8 d. DEM data (ASTER GDEM) were sourced from the Geospatial Data Cloud Platform (http://www.gscloud.cn), with a spatial resolution of 90 × 90 m.

### Research methods

#### Soil physicochemical property indicators and surface factors

Soil physicochemical property indicators include soil bulk density (BD), soil pH, soil clay (clay), silt (silt), and sand (sand), and were obtained from experimental data. Surface factors consist of 11 factors describing vegetation, atmosphere, landscape, and ecological environment: vegetation index (NDVI), net primary productivity (NPP), surface moisture (WET), soil salinity index (SSI), urban building index (IBI), bare soil index (BSI), boundary density (ED), maximum patch index (LPI), annual rainfall (Pre), annual mean temperature (Tem), and soil erosion (RUSLE).

#### Determination of soil factors

##### Soil data

The samples were divided into two layers (0–20 and 20–50 cm). Soil TN and TC were measured by a VaroELШ elemental analyzer (He, 2017); soil particle size was measured by a Mastersize 2000 laser particle sizer (Li, 2018); soil bulk density was measured by the cutting-ring method (He, 2017); soil pH was measured by a pH meter (Feng et al., 2011).

#### Calculation of ecological indicators

Soil moisture calculation (Guo et al., 2021):

$$\begin{aligned}\mathrm{WET}\;(MOD09A1)=&\;0.1147{\rho }_{\mathrm{red}}+0.2489{\rho }_{\mathrm{nir}1}\\&+0.2408{\rho }_{\mathrm{blue}}+0.3132{\rho }_{\mathrm{green}}\\&-0.3122{\rho }_{\mathrm{nir}2}-0.6416{\rho }_{\mathrm{swir}1}\\&-0.5087{\rho }_{\mathrm{swir}2}\end{aligned}$$

Urban building index calculation:

$$\mathrm{IBI}\;(\mathrm{MOD}09{A}1)=\frac{\frac{2{\rho }_{\mathrm{swir}1}}{{\rho }_{\mathrm{swir}1}\,+\,{\rho }_{\mathrm{nir}1}}-\left(\frac{{\rho }_{\mathrm{nir}1}}{{\rho }_{\mathrm{nir}1}\,+\,{\rho }_{\mathrm{red}}}+\frac{{\rho }_{\mathrm{green}}}{{\rho }_{\mathrm{green}}\,+\,{\rho }_{\mathrm{swir}1}}\right)}{\frac{2{\rho }_{\mathrm{swir}1}}{{\rho }_{\mathrm{swir}1}\,+\,{\rho }_{\mathrm{nir}1}}+\left(\frac{{\rho }_{\mathrm{nir}1}}{{\rho }_{\mathrm{nir}1}\,+\,{\rho }_{\mathrm{red}}}+\frac{{\rho }_{\mathrm{green}}}{{\rho }_{\mathrm{green}}\,+\,{\rho }_{\mathrm{swir}1}}\right)}$$

Bare soil index calculation:

$$\mathrm{SI}\;(\mathrm{MOD}09{A}1)=\frac{({\rho }_{\mathrm{swir}1}\,+\,{\rho }_{\mathrm{red}})-({\rho }_{\mathrm{nir}1}\,+\,{\rho }_{\mathrm{blue}})}{({\rho }_{\mathrm{swir}1}\,+\,{\rho }_{\mathrm{red}})\,+\,({\rho }_{\mathrm{nir}1}+{\rho }_{\mathrm{blue}})}$$

Soil salinity index calculation (Yang et al., 2021):

$${SSI}({Landsat}8)=\sqrt{{\rho }_{\mathrm{blue}}\times {\rho }_{\mathrm{red}}}$$

where *ρ*_red_, *ρ*_nir1_, *ρ*_blue_, *ρ*_green_, *ρ*_nir2_,* ρ*_swir1_, and* ρ*_swir2_ represent the ground object reflectivities of the red band, near-infrared band 1, blue band, green band, near-infrared band 2, mid-infrared band 1, and mid-infrared band 2 of Landsat and MODIS satellite data, respectively.

#### Soil erosion calculation

The RUSLE soil loss equation was used to quantitatively evaluate the soil erosion in the study area in 2018. The calculation formula is as follows (Wei et al., 2021):

$$A = R\;\;K\;\;L\;\;S\;\;C\;\;P$$

where *A* is the soil erosion modulus (t/(hm^2^·a)); *R* is the rainfall erosivity factor ((MJ·mm)/(hm^2^·a)); *K* is the soil erodibility factor (t·hm^2^·h)/(hm^2^·hm^2^); LS is the slope length and slope factor, dimensionless; *C* is the vegetation cover management factor, dimensionless, and value range of 0–1; the water and soil conservation measure factor *P* is related to the land use type, dimensionless, and the value range is 0–1. *R* reflects the impact of rainfall on soil erosion. The rainfall observation data of meteorological stations distributed in Qinghai Province was sorted to obtain the monthly average rainfall and annual average rainfall in the study area, and the rainfall at each station was calculated. The rainfall erosivity factor of the whole study area was obtained by random forest interpolation, and the interpolation accuracy was above 0.8.

#### Calculation of soil TC and TN changes

The measured soil TN and TC data were used to assess horizontal and vertical distribution characteristics. △ TC and △ TN represent the changes in soil TC and TN in different soil layers, respectively, and were calculated using the following equations (Dahai & Yuan, 2020):

$${\Delta TC}=\;^{{\left({{TC}}_{y}-{{TC}}_{x}\right)}}\bigg/_{{{TC}}_{x}}\times 100{\%}$$

$${\Delta TN}=\;^{{\left({{TN}}_{y}-{{TN}}_{x}\right)}}\bigg/_{{{TN}}_{x}}\times 100\%$$

where TC_*y*_, TC_*x*_, TN_*y*_, and TN_*x*_ represent the carbon and nitrogen values of the soil layers at 0–20 cm (*y*) and 20–50 cm (*x*), respectively, and △TC and △TN represent the difference between soil TN and TC in the two soil layers, respectively.

#### Analysis methods

Correlation analysis was used in SPSS to analyze the single influencing factors of soil TC and TN, and one-way analysis of variance (ANOVA) was used for significance analysis. The factors were divided into quantiles of [0–20], [20–40], [40–60], [60–80], and [80–100], and then the RDA in CANOCO 5 software was used to perform synergy analysis on the influencing factors.

## Results

### Distribution characteristics of soil carbon and nitrogen content

#### Statistical analysis of soil TC and TN

As shown in Table 1, the average soil carbon and nitrogen values in the surface layer were greater than those in the deep layer, indicating that the soil carbon and nitrogen values show a decreasing trend. The coefficients of variation were all lower than 48–60%, so they belonged to medium-intensity variation. The skewness and kurtosis of soil carbon and nitrogen were both greater than 0, indicating that the kurtosis is biased towards smaller values and the distribution is relatively sharp.

**Table 1:** Descriptive statistics of soil TC and TN

Type	Minimum (g/kg)	Maximum (g/kg)	Mean (g/kg)	Standard deviation	Skewness	Kurtosis	Coefficient of variation %
0–20 cmTC	7.88	200.49	65.87	38.90	1.14	0.92	59.06
0–20 cmTN	0.63	15.23	5.28	2.96	0.91	0.80	56.03
20–50 cmTN	0.81	10.69	3.43	1.70	1.56	3.89	49.41
20–50 cmTC	13.56	136.86	45.44	22.03	1.64	3.65	48.48

#### Characteristics of soil TC and TN in different community types

**Fig. 3:**
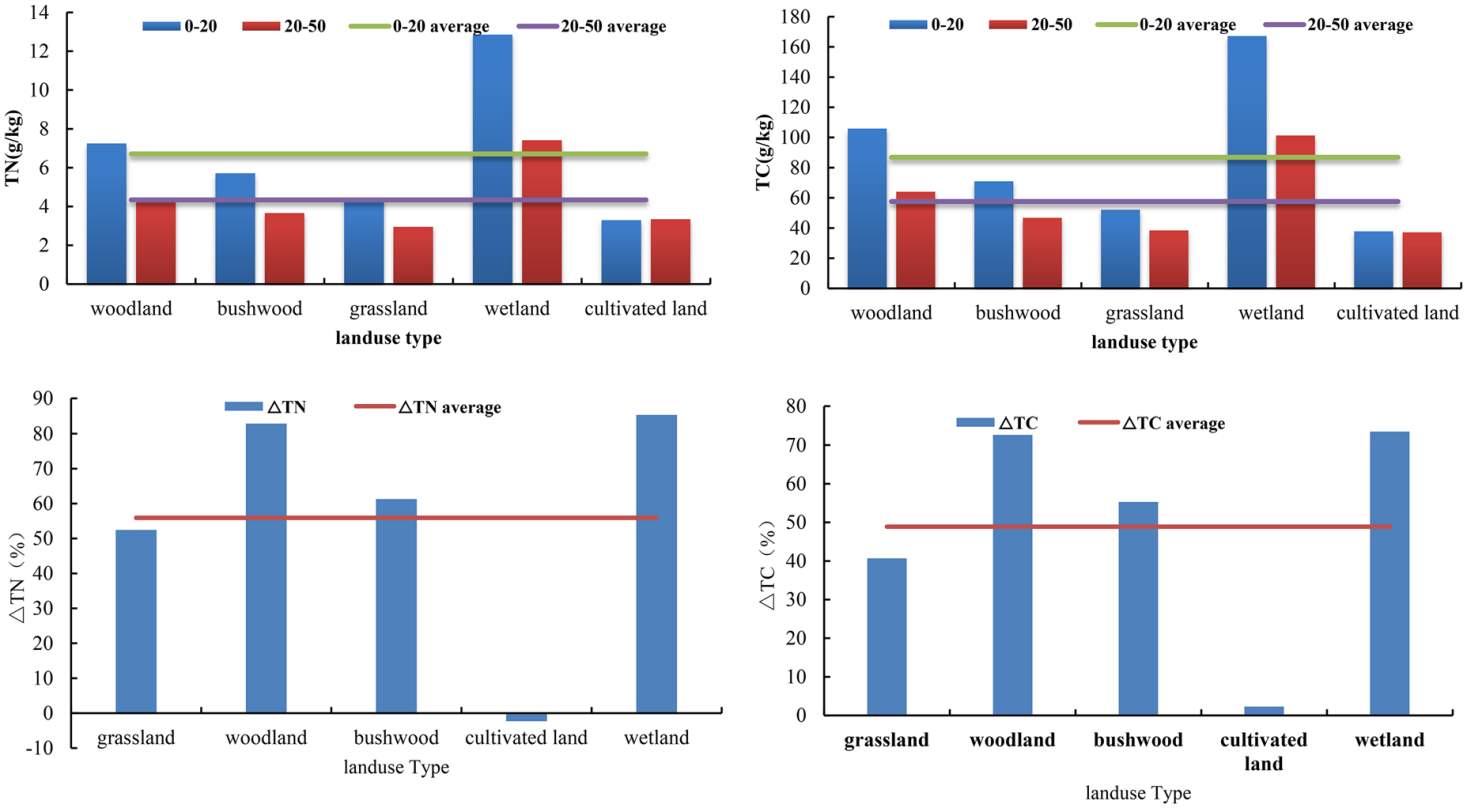
Changes in TN, TC, △TC, and △TN in the study area and different land use types

The average values of TC and TN in soil at 0–20 cm in the study area were 86.55 and 6.7 g/kg, respectively; at 20–50 cm, the average values were 57.58 and 4.34 g/kg, respectively. TC and TN decrease from the surface layer to the deep layer. Wetlands had the highest TC and TN among different communities, followed by woodland, shrubland, grassland, and cultivated land. The average values of TC and TN were all greater than 0, 48.89%, 55.92%, and 6.46%, respectively. The TN of cultivated land was less than 0, but TN values were all greater than 0 for the other samples. The ordering of TC and TN by size in various communities was consistent with that of TC and TN (Fig. 3).

### Spatial distribution characteristics of soil carbon and nitrogen

**Fig. 4:**
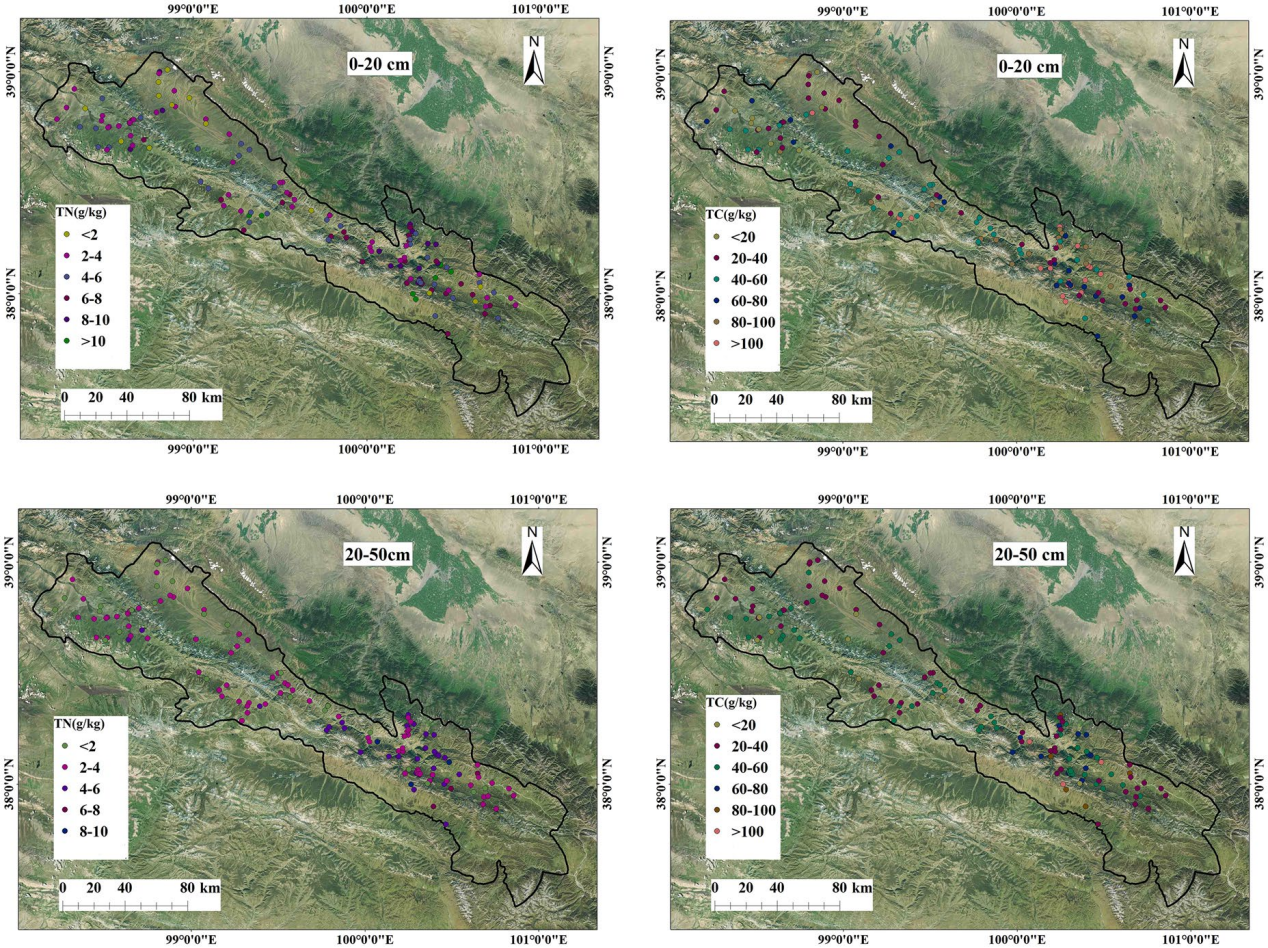
Spatial distribution of soil TC and TN

**Fig. 5:**
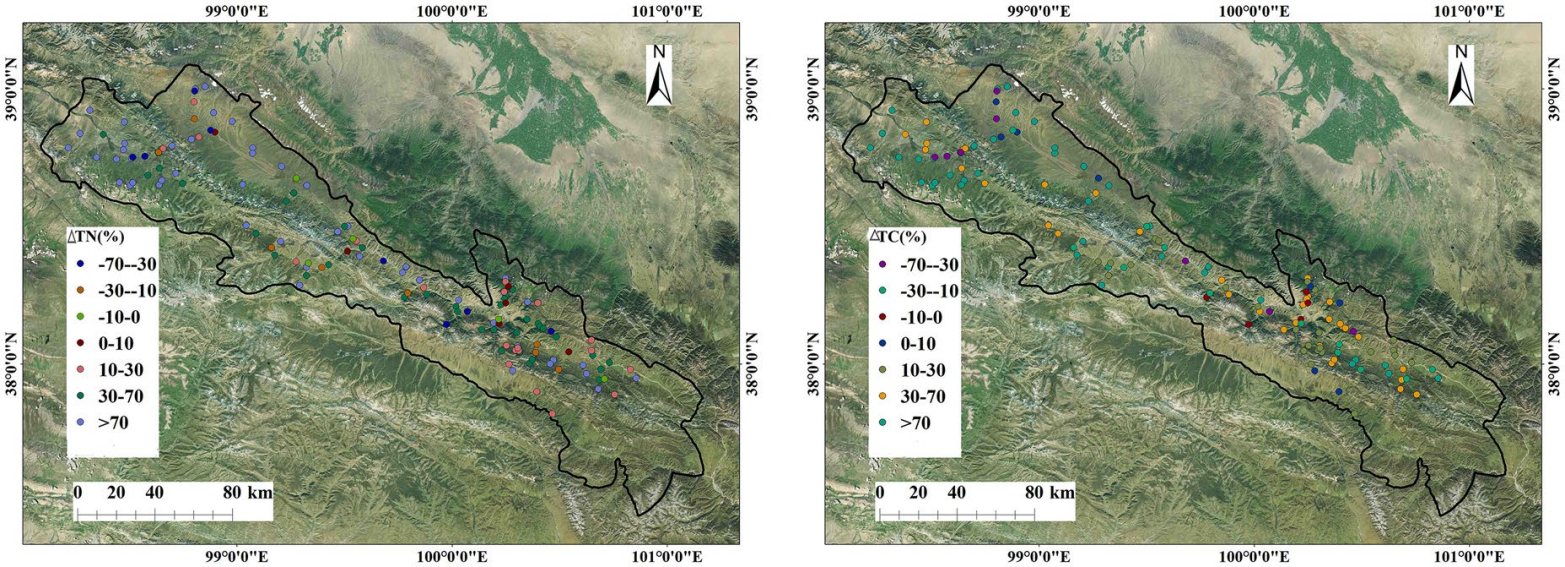
Spatial distribution of soil △TC and△TN

As shown in Fig. 4, the spatial differences in soil TC and TN in the whole study area were relatively significant. The high-value areas were clustered in the southeast of Qilian County, and the low-value areas were clustered in the northwest of Qilian County, and △TN exhibited the opposite trend (Fig. 5). These differences were due to the large difference in nitrogen content between the surface layer and the deep layer.

As shown in Table 2, soil TC and TN in wetland at 0–20 cm were significantly higher than in other land use types, and soil TC and TN at 0–20 cm were significantly different and were in the order from highest to lowest of wetland, woodland, shrubland, and cultivated land. The soil TC and TN at 20–50 cm in the wetland and woodland were significantly different from those of shrubs, cultivated land, and grassland, but there was no significant difference in soil TC and TN between shrubland, cultivated land, and grassland. △TC and △TN decreased from top to bottom, without any significant difference.

**Table 2:** Soil carbon and nitrogen characteristics of different land use types

Land use type	0–20 cmTC	0–20 cmTN	20–50 cmTC	20–50 cmTN	△TC	△TN
Grassland	52.16 ± 2.73cd	4.43 ± 0.26 cd	38.49 ± 1.54c	2.96 ± 0.14c	− 0.13 ± 0.05ab	− 0.20 ± 0.05ab
Cultivated land	37.79 ± 4.07d	3.29 ± 0.44d	37.15 ± 1.90c	3.35 ± 0.24bc	0.02 ± 0.11a	0.07 ± 0.11a
Bushwood	70.96 ± 6.34c	5.71 ± 0.49bc	46.82 ± 3.73c	3.66 ± 0.29bc	− 0.29 ± 0.05ab	− 0.32 ± 0.04b
Woodland	106.02 ± 8.84b	7.25 ± 0.45b	64.05 ± 5.81b	4.31 ± 0.36b	− 0.38 ± 2.73b	− 0.39 ± 0.05b
Wetland	167.34 ± 7.57a	12.86 ± 0.92a	101.39 ± 11.41a	7.41 ± 1.08a	− 0.39 ± 0.07b	− 0.43 ± 0.06b

If the same indicator has the same letter in different ecosystems, it indicates that there is no significant difference, otherwise there is a significant difference

### Analysis of correlation between soil carbon and nitrogen and soil factors and environmental factors

#### Analysis of correlation between soil carbon and nitrogen and soil factors

Table 3 shows the correlation coefficients of different physical and chemical properties of soil in different soil layers. The correlation coefficients of BD and clay progressively decreased from the surface layer to the deep layer, while the correlation coefficients of silt, sand, and pH increased progressively. The order of the correlation coefficients was BD > silt > clay > sand > pH. Soil TC and TN were strongly negatively correlated with BD. Clay was significantly positively correlated with TC and TN in soil at 0–20 cm but not at 20–50 cm. Silt was significantly correlated with soil TC and TN. Sand and pH were significantly negatively correlated with soil TC and TN.

**Table 3:** Analysis of carbon and nitrogen related factors

Type	BD	Clay	Silt	Sand	pH
0–20TC	− 0.73**	0.36**	0.41**	− 0.33**	− 0.19*
0–20TN	− 0.72**	0.33**	0.35**	− 0.27**	− 0.23**
20–50TC	− 0.66*	0.13	0.47**	− 0.45**	− 0.2 8**
20–50TN	− 0.67**	0.10	0.40**	− 0.38**	− 0.31**

*At the 0.05 level (two tailed), the correlation was significant

**At the 0.01 level (two-tailed), the correlation was significant

#### Analysis of correlation between soil carbon and nitrogen and environmental factors

As shown in Table 4, TC and TN of soil at 0–20 cm were significantly correlated with WET, Pre, NPP, SSI, NDVI, BI, LPI, and ED. WET, SSI, LPI, and ED were negatively correlated with soil carbon and nitrogen, and the other factors were positively correlated with soil carbon and nitrogen. WET, LPI, and ED were more closely correlated with soil TC, and WET, Pre, and NDVI were more closely correlated with soil TN. TC and TN in soil at 20–50 cm were more correlated with Tem than in the 0–20 cm soil layer, and WET, Pre, and ED were more closely correlated with TN. There was no significant correlation of △TC with Tem, IBI, RUSLE, and ED, but the other factors showed a significant correlation. There was little correlation of △TN had no significant correlation with any factors.

**Table 4:** Correlation between soil carbon and nitrogen and environmental factors

Type	WET	Tem	Pre	NPP	SSI	NDVI	IBI	BSI	RUSLE	LPI	ED
0–20TC	− 0.45**	0.13	0.36**	0.22**	− 0.30**	0.38**	0.06	0.20*	0.00	− 0.45**	− 0.41**
0–20TN	− 0.44**	0.09	0.39**	0.27**	− 0.24**	0.42**	0.10	0.17*	0.01	− 0.37**	− 0.34**
20–50TN	− 0.37**	0.19*	0.39**	0.29**	− 0.21*	0.36**	0.16	0.17*	− 0.05	− 0.36**	− 0.37**
20–50TC	− 0.32**	0.21*	0.31**	0.18*	− 0.29**	0.27**	0.14	0.21*	0.01	− 0.40**	− 0.39**
△TC	0.30**	− 0.05	− 0.24**	− 0.30**	0.22*	− 0.45**	− 0.03	− 0.18*	− 0.04	0.21*	0.15
△TN	0.25**	0.03	− 0.19*	− 0.22**	0.19*	− 0.35**	− 0.02	− 0.16	− 0.10	0.15	0.09

*At the 0.05 level (two-tailed), the correlation was significant; **At the 0.01 level (two-tailed), the correlation was significant

#### Correlation between influencing factors and sorting axis

The RDA analysis of soil carbon and nitrogen from 0 to 20 cm is presented in Table 5. BD had the closest relationship with the first ranking axis, with a correlation coefficient of 0.73, and also had a close correlation with WET, LPI, ED, and silt, with all correlation coefficients above 0.4. This indicates that soil TC and TN mainly reflect the gradient changes in BD, WET, LPI, ED, and silt on the first axis. With the decrease in BD, soil carbon and nitrogen increase. The correlation between pH and NPP and the second-ranking axis was the highest, 0.17 and − 0.17, indicating that the second axis reflects the gradient change in pH and NPP with soil TC and TN. In the RDA analysis of TC and TN in soil at 20–50 cm, BD had the closest relationship with the first ranking axis, with a correlation coefficient up to − 0.66, and had a high correlation with LPI, silt, and sand, with all correlation coefficients above 0.4. This indicates that soil TC and TN primarily reflect the gradient changes in BD, LPI, silt, and sand on the first axis. Soil TC and TN increased with the decrease in BD. NDVI had the highest correlation with the second sorting axis, at 0.23, suggesting that this axis basically reflects the gradient change in NDVI with soil TC and TN.

**Table 5:** Coefficients of correlations between the first two sorting axes of RDA at different soil depths and influencing factors

Influencing factors	0–20 cm		20–50 cm	
	Axis 1	Axis 2	Axis 1	Axis 2
BD	0.73	0.04	− 0.66	0.06
Clay	− 0.36	0.11	0.12	0.05
Silt	− 0.40	0.16	0.47	0.18
Sand	0.33	− 0.12	− 0.45	− 0.17
pH	0.19	0.17	− 0.28	0.16
WET	0.45	0.01	− 0.32	0.12
Tem	− 0.13	0.03	0.21	− 0.01
Pre	− 0.36	− 0.10	0.31	− 0.16
NPP	− 0.22	− 0.17	0.18	− 0.20
SSI	0.30	− 0.14	− 0.29	− 0.13
NDVI	− 0.38	− 0.15	0.27	− 0.23
IBI	− 0.06	− 0.09	0.14	− 0.01
BSI	0.23	0.09	0.21	0.07
RUSLE	0.00	− 0.02	0.00	0.11
LPI	0.45	− 0.12	− 0.40	− 0.04
ED	0.41	− 0.08	− 0.39	0.00

### Synergistic effects of soil physical properties and environmental factors

#### Synergistic effect of soil factors

**Fig. 6:**
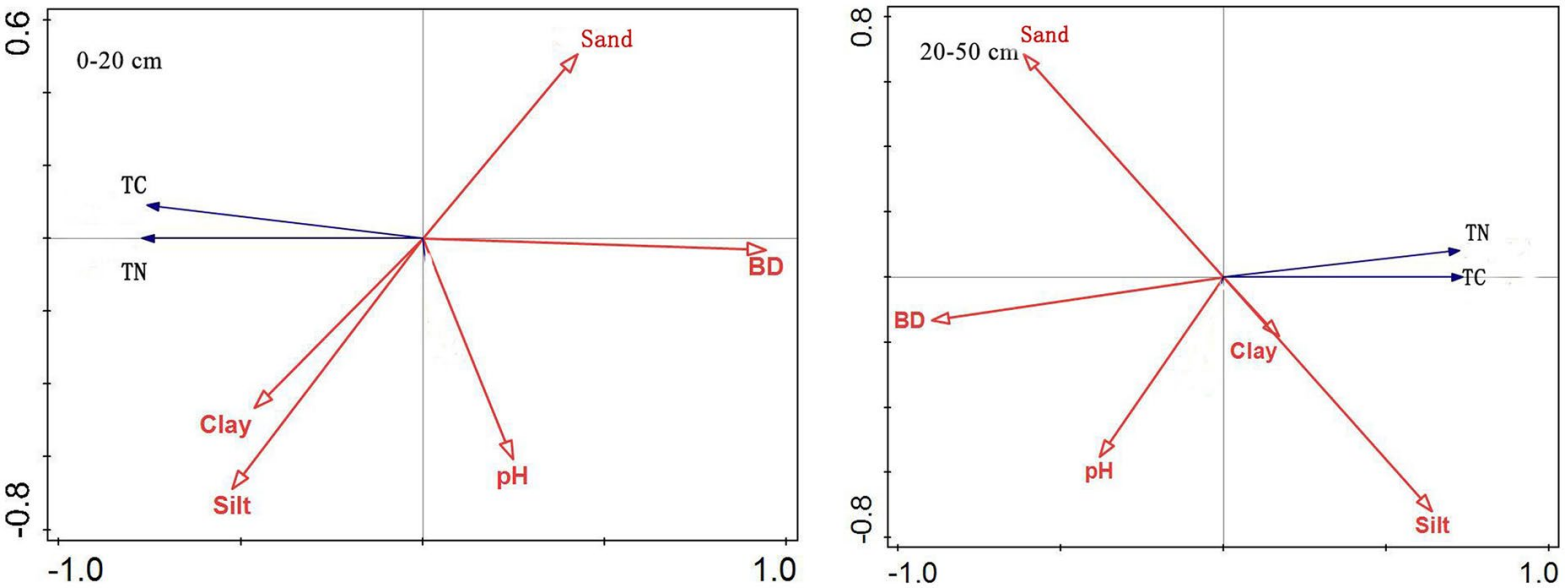
RDA ranking of soil carbon and nitrogen and soil factors at 0–20 and 20–50 cm

According to the RDA ranking analysis (Fig. 6), all standard axes were found to be statistically significant. This figure shows the impact of soil properties on soil total carbon and nitrogen. The positions of TN and TC in the two layers of soil were close, indicating a strong positive correlation. In soil layers at 0–20 and 20–50 cm, high soil TN and TC were correlated with high clay and silt and low BD, but less sensitive to sand and pH. This is consistent with the previous analysis of the factors affecting carbon and nitrogen in the Qilian Mountains: the correlation between clay particles and soil C and N gradually decreased with the increase of soil depth, and soil BD had a significant negative correlation with soil C and N (Yu, 2020; Yuan, 2019).

**Fig. 7:**
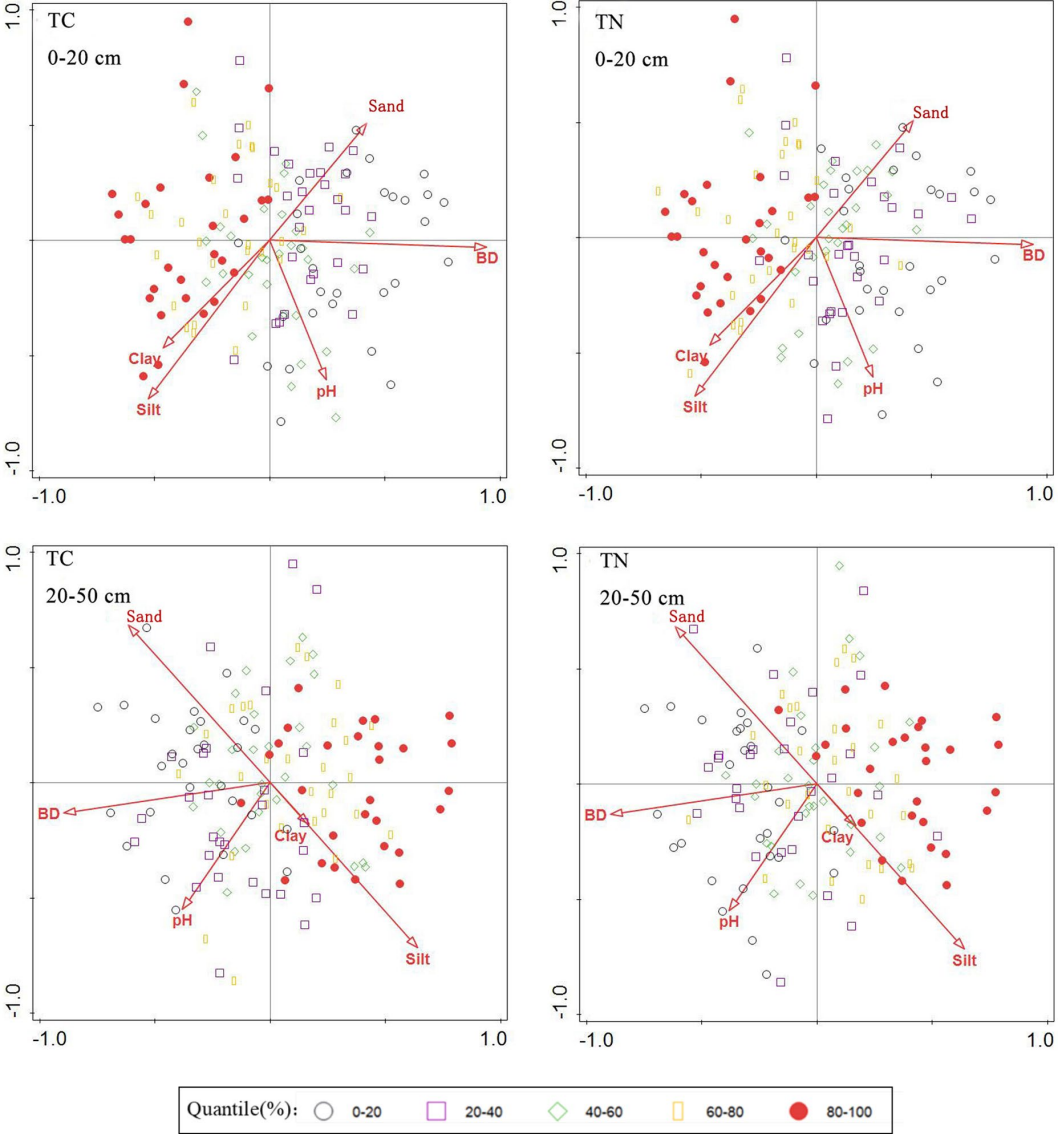
RDA ranking of soil factor sampling points in different soil layers

Figure 7 depicts the RDA ranking of soil factor sampling points in different soil layers. This figure shows the impact of soil properties on different soil carbon and nitrogen value ranges. The high values of TC and TN in soil at 0–20 cm are generally shown on the left side of the diagram, while the low values of TC and TN in soil are shown on the right side. High soil TC and TN sampling points are found in areas with higher clay and silt and lower BD, pH, and sand. The high values of soil TC and TN sampling points appear on the right side of the diagram at 20–50 cm, and the low values of soil TC and TN appear on the left side of the diagram. High soil TC and TN sampling points are distributed in areas with higher silt and lower clay, BD, pH, and sand.

#### Synergistic effect of environmental factors

**Fig. 8:**
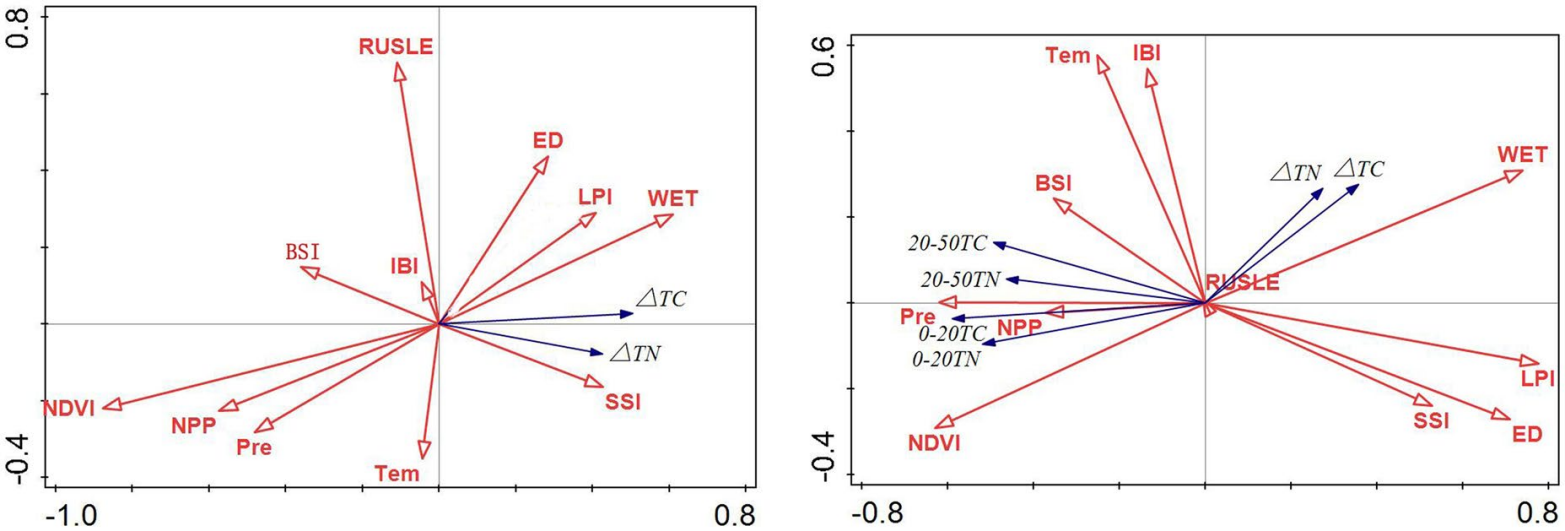
RDA ranking of soil carbon and nitrogen and environmental factors at 0–20 and 20–50 cm

According to the ranking diagram of soil TC, TN, and environmental factors (Fig. 8), the high values of sampling points of TN and TC in soil at 0–20 and 20–50 cm correlate with higher Pre, NPP, NDVI and lower WET, LPI, ED, and SSI. The diagram on the right shows the RDA ranking of △TC and △TN and environmental factors, revealing that △TC and △TN are relatively close to each other and are located at positions with higher WET, LPI, ED, and SSI and lower values for other environmental factors.

**Fig. 9:**
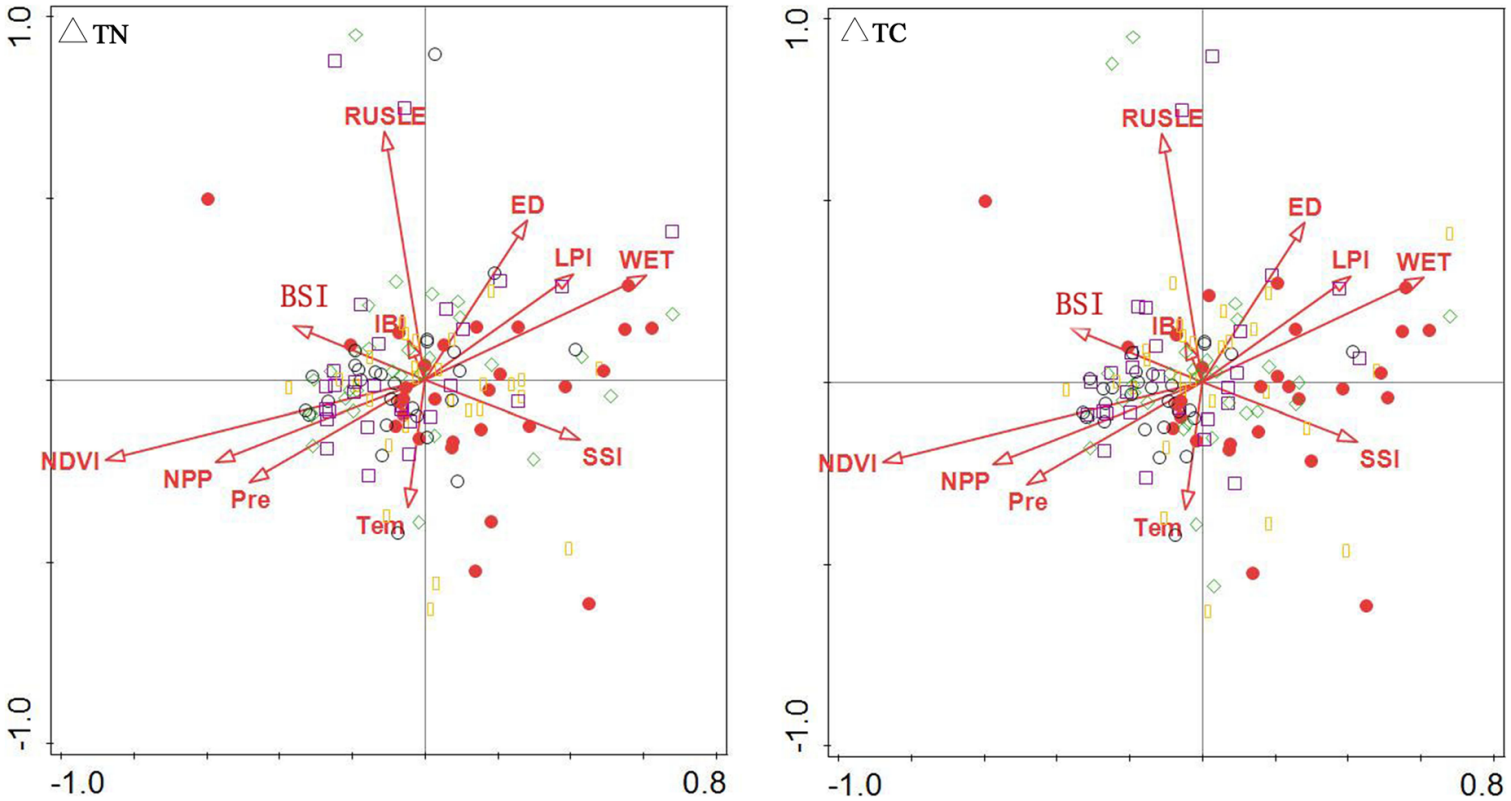
RDA ranking of surface factors and sampling points of ΔTC and ΔTN

As shown in Fig. 9, the spatial trends of △TC and △TN to the right were significant. This figure shows the impact of environmental factors on the range of carbon and nitrogen differences in different soils. The sampling points with high △TC and △TN were chiefly distributed in areas with higher WET, LPI, ED, and SSI, with a low correlation with the spatial position of RUSLE, IBI, and Tem.

**Fig. 10:**
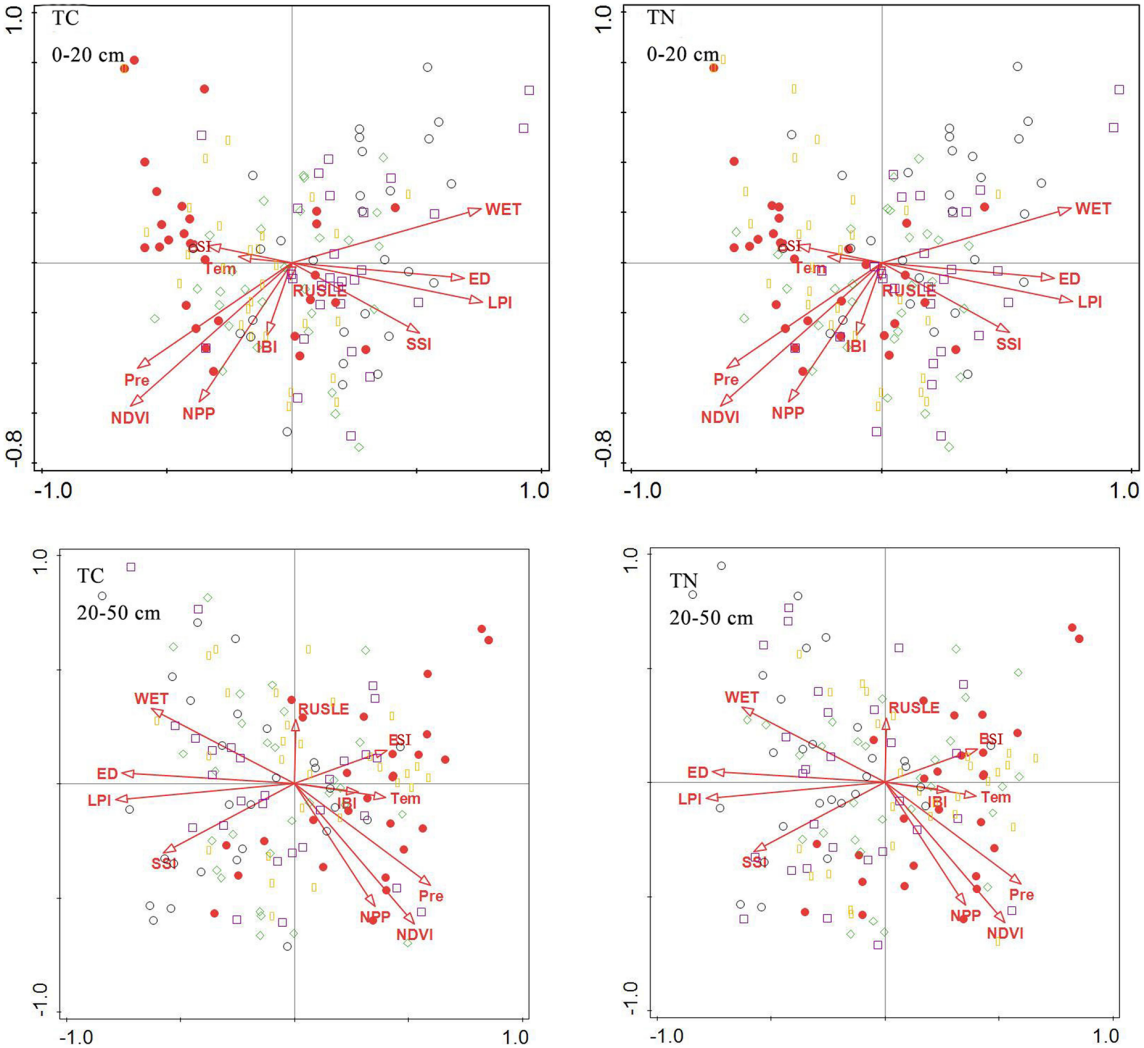
RDA ranking of sampling points of soil TC and TN land surface factors

At 0–20 cm, the soil TC and TN were significantly inclined to the left in Fig. 10. This figure shows the impact of environmental factors on different soil carbon and nitrogen value ranges. The high values of soil TC and TN at the sampling points were correlated with higher TEM, IBI, Pre, NDVI, and NPP. However, at 20–50 cm, soil TC and TN space were significantly inclined to the right, and the high values of soil TC and TN at the sampling points correlated with higher BSI, IBI, TEM, NDVI, and NPP.

## Discussion

### Differences in soil TC and TN distribution in different communities

Soil nutrients exhibit spatial heterogeneity due to variation in factors such as topography, climate, landscape, and human activities. In this study, the horizontal distribution of soil TC and TN exhibited obvious variations in spatial distribution, with higher values in the southeast compared to those in the northwest. This is because the southeastern zone is dominated by forest land and shrubs, and the northwestern zone is dominated by grassland and unused land. The soil samples in areas of wetlands, woodlands, and shrubs had the largest TC and TN values. These high values are explained by the soil moisture status and temperature significantly affecting the nitrogen mineralization process by altering the microbial community and activities (Wang et al., 2006). Wilson and Tiley (1998) studied soil nitrogen mineralization in wetland ecosystems and showed that soil moisture and temperature have a significant impact on the rate of soil nitrogen mineralization, with high temperature and drought promoting soil mineralization. In this study, samples were taken in August, a time with abundant rain and highest soil moisture content. There is a significant positive correlation between soil carbon and nitrogen and rainfall (Table 3), and wetland areas have the largest annual rainfall (Table 6). Therefore, wetlands have the largest soil carbon and nitrogen among the different areas tested. Forest land and shrubs are highly covered with vegetation, generally at high altitudes, with little human disturbance and well-developed root systems in the soil. Dead trees, fallen leaves, roots, and microbial activities can increase the soil’s organic carbon content (Hou et al., 2021). When the soil water content is high, the anaerobic decomposition of organic matter is inhibited, resulting in the accumulation of organic carbon (Hu et al., 2018). This can also limit the activities of soil microorganisms and inhibit the mineralization and decomposition of organic nitrogen (Bai et al., 2006; Zhao et al., 2016). The low content of carbon and nitrogen in the grassland and cultivated land soil samples may be because the cultivated land is mostly dry land. In dry areas, mineralization of the soil is promoted, impeding the accumulation of carbon. The study area is used for grazing, and the grassland can be easily disturbed and easily damaged.

**Table 6:** Annual rainfall in different ecosystems

Land use type	Cultivated	Bushwood	Woodland	Wetland	Grassland
Annual precipitation (mm)	537.14	571.62	563.06	580.4	511.53

There were higher TC and TN values in the 0–20 cm soil samples compared to those at 20–50 cm. This is because the surface layer contains more animal and plant residues and more humus, while the deep soil has more roots that impede the decomposition of organic carbon. The distribution of ΔTC and ΔTN is similar to that of soil TC and TN.

Based on RDA analysis from different perspectives, this study explored the contribution rates of factors. As shown in Table 7, the three most influential factors of surface layer soil at 0–20 cm are BD, pH, and clay, and the three most influential factors at 20–50 cm are BD, silt, and pH. LPI, WET, and SSI are the environmental factors that make the highest contribution to the vertical distribution of soil carbon and nitrogen. The three most influential factors for the vertical distribution are NDVI, RUSLE, and IBI, and the most influential factors for the horizontal distribution are LPI, WET, and NPP.

**Table 7:** Contribution rates of influencing factors

	0–20	20–50		All layers	Across layers
Soil factor	Contribution %	Contribution %	Environmental Factors	Contribution %	Contribution %
BD	88.8	79.9	LPI	58.8	0.3
Clay	4.3	1.3	WET	18	2.7
pH	6.1	7.7	SSI	8.9	0.5
Silt	0.5	11	NDVI	1.7	72.2
Sand	0.3	0.1	NPP	5.6	0.6
			Tem	3.4	2.1
			BI	1.7	2.2
			ED	1	2.1
			RUSLE	0.4	9.4
			IBI	0.3	7.4
			Pre	0.1	0.4

### Influencing factors of soil TC and TN changes

For the physical and chemical properties of soil, the correlation coefficients of BD and clay decreased from the surface layer to the deep layer, and those of silt, sand, and pH increased. This may be because the surface soil has higher bulk density and a higher proportion of clay particles and is more easily disturbed by human factors, giving rise to a progressive decrease. The high values of soil TC and TN are distributed in areas with higher clay and silt and lower BD, pH, and sand. BD is negatively correlated with soil carbon and nitrogen because the soil organic matter has important effects on soil aggregates and mineral structure and composition (Zhang et al., 2022). Low soil bulk density, large pores, fine texture, and enhanced water infiltration capacity lead to reduced surface runoff and high soil water content, contributing to the accumulation of soil nutrients (Qu, 2019). Soil pH affects the capacity to fix and accumulate carbon and nitrogen by affecting the activities of soil microorganisms (Bai et al., 2003). In an alkaline environment, the activities of microorganisms are inhibited, resulting in a decrease in carbon and nitrogen content.

The correlation analysis results are consistent with the RDA analysis results. For the environmental factors, the highest values of soil TN and TC sampling points in soil layers at 0–20 cm and 20–50 cm correlate with higher Pre, NPP, NDVI, and IBI values and lower WET, LPI, ED, and BSI values. Under natural conditions, vegetation is an important source of soil organic carbon, and different vegetation types will affect the spatial distribution of soil organic carbon and total nitrogen. Different vegetation types have different biomass and can differ in the degrees of decay of litter and residues, resulting in differences in soil input and output organic matter. Fang (2010) proposed that soil organic carbon in frigid grassland was significantly and positively correlated with precipitation, which is consistent with the results of this study. Increased precipitation contributes to plant growth and promotes the accumulation of surface biomass and the return of organic matter, thus elevating soil carbon and nitrogen content (Zhang et al., 2021). Smaller LPI indicates a higher degree of landscape fragmentation, reduced biodiversity, and soil fertility. However, as shown in Table 8, although the largest LPI in the study area occurs in grassland, the soil TC and TN contents of grassland are relatively low in this area due to the comprehensive influence of other ecological environments and physicochemical properties (Fig. 3). As a result, there is a negative correlation between the LPI and TC or TN. Lower ED correlated with a lower degree of landscape fragmentation, higher biodiversity, and higher relative soil carbon and nitrogen content. Lower BSI indicates a smaller area of bare soil and larger area of vegetation coverage, promoting the storage of soil carbon and nitrogen. The surface temperature is generally higher than the air temperature. For frigid vegetation, low temperature is suitable, and high temperature may impede the growth of vegetation. As a result, there is a negative correlation between BSI and TC or TN.

**Table 8:** LPI values of different ecosystems

Grassland	Cultivated	Bushwood	Woodland	Wetland
21.81	1.02	1.24	1.24	3.26

### Prospects

In this study, the spatial distribution and influencing factors of soil TC and TN were analyzed. The uneven distribution of sample points and limited collection of sample points in different ecosystems due to accessibility impacted the research results. The study of influencing factors included few soil physical and chemical properties due to insufficient data for available phosphorus, available potassium, and microorganisms, but future work should investigate the contributions of these factors to more fully understand the influencing factors of soil TC and TN. Strengthening the study of soil physical and chemical properties can not only provide more specific and detailed basic data for the implementation of ecological engineering in the study area, but it can also promote the smooth development of the project and provide a theoretical foundation for future research.

## Conclusions

This study determined the variation law of TC and TN contents in soil in the horizontal and vertical directions and analyzed the soil TC and TN in different types of communities. The individual and synergistic effects of soil TC and TN spatial patterns were assessed using correlation analyses and RDA ranking methods for 16 factors in four categories: soil, vegetation, landscape, and ecological environment. The results showed variation in the characteristics of soil TC and TN in the source region of Heihe River for different types of communities and at different soil depths. The soil TC and TN values were in the order of wetland > forest > shrub > grassland > arable land. The surface soil layer and deep soil layer analysis results were consistent across ecosystems. In terms of soil factors, areas with higher clay and silt and lower BD, pH, and sand have higher soil TC and TN values. The correlation analysis results match the RDA analysis results. Higher soil TN and TC values were associated with higher Pre, NPP, NDVI, and IBI values and lower WET, LPI, ED, and BSI values. Among soil factors, BD and silt are most closely correlated with TC and TN in each layer; NDVI, RUSLE, and IBI surface factors influence vertical distribution; and LPI, WET, and NPP surface factors influence horizontal distribution.

## Data Availability

The datasets used and/or analyzed during the current study are available from the corresponding author upon reasonable request.
